# Psychosocial aspects and mental health burden of pediatric MASLD

**DOI:** 10.3389/fendo.2026.1916057

**Published:** 2026-08-24

**Authors:** Vanishri Ganakumar, Abhinay Mahaling, Manjunath Goroshi, Vikrant Ghatnatti

**Affiliations:** Department of Endocrinology, Jawaharlal Nehru Medical College, KLE Academy of Higher Education and Research (KAHER), Belgaum, India

**Keywords:** biopsychosocial model, childhood obesity, depression, mental health, non-alcoholic fatty liver disease, pediatric MASLD, quality of life, eating disorders

## Abstract

Metabolic dysfunction–associated steatotic liver disease (MASLD) is the most common chronic liver disease of childhood and the hepatic component of a systemic metabolic and endocrine disorder linked to obesity and insulin resistance. Its psychological and social dimensions, however, remain under-recognized relative to its metabolic features. Affected children carry a substantial mental-health burden: elevated rates of depression, anxiety, disordered eating, and impaired quality of life that both reflect and aggravate the underlying disease. This mini review uses the biopsychosocial model to summarize current understanding of this burden in pediatric MASLD. We outline the bidirectional neuroendocrine and inflammatory mechanisms that link metabolic dysfunction with psychological distress, including cytokine signalling, leptin and adipokine dysregulation, hypothalamic–pituitary–adrenal axis activation, and the gut–brain axis. We then consider the social determinants and system-level barriers that shape outcomes, practical approaches to screening and integrated multidisciplinary management, and the psychosocial considerations raised by emerging pharmacotherapies such as glucagon-like peptide-1 receptor agonists. We highlight areas of ongoing debate: notably the tension between weight-centred intervention and the risk of precipitating disordered eating, and the neuropsychiatric monitoring of new pharmacological agents, together with the principal research gaps and likely future directions. Recognizing pediatric MASLD as a biopsychosocial condition, and embedding mental-health assessment within routine metabolic care, may improve both hepatic and psychological outcomes in this growing population.

## Introduction

Metabolic dysfunction–associated steatotic liver disease (MASLD) is now a leading cause of chronic liver disease in children and has increased in step with rising pediatric obesity. The change in nomenclature from non-alcoholic fatty liver disease (NAFLD) to MASLD in 2023 was motivated, in part, by concerns about stigma: in the Delphi consensus process, 66% and 61% of experts considered the terms “fatty” and “non-alcoholic,” respectively, to be stigmatizing (1). In a condition closely associated with childhood obesity, terminology may itself shape how children and families experience the illness. MASLD is therefore used as the preferred term in this review, while acknowledging that most underlying pediatric studies were conducted under the earlier NAFLD terminology.

**Figure 1:**
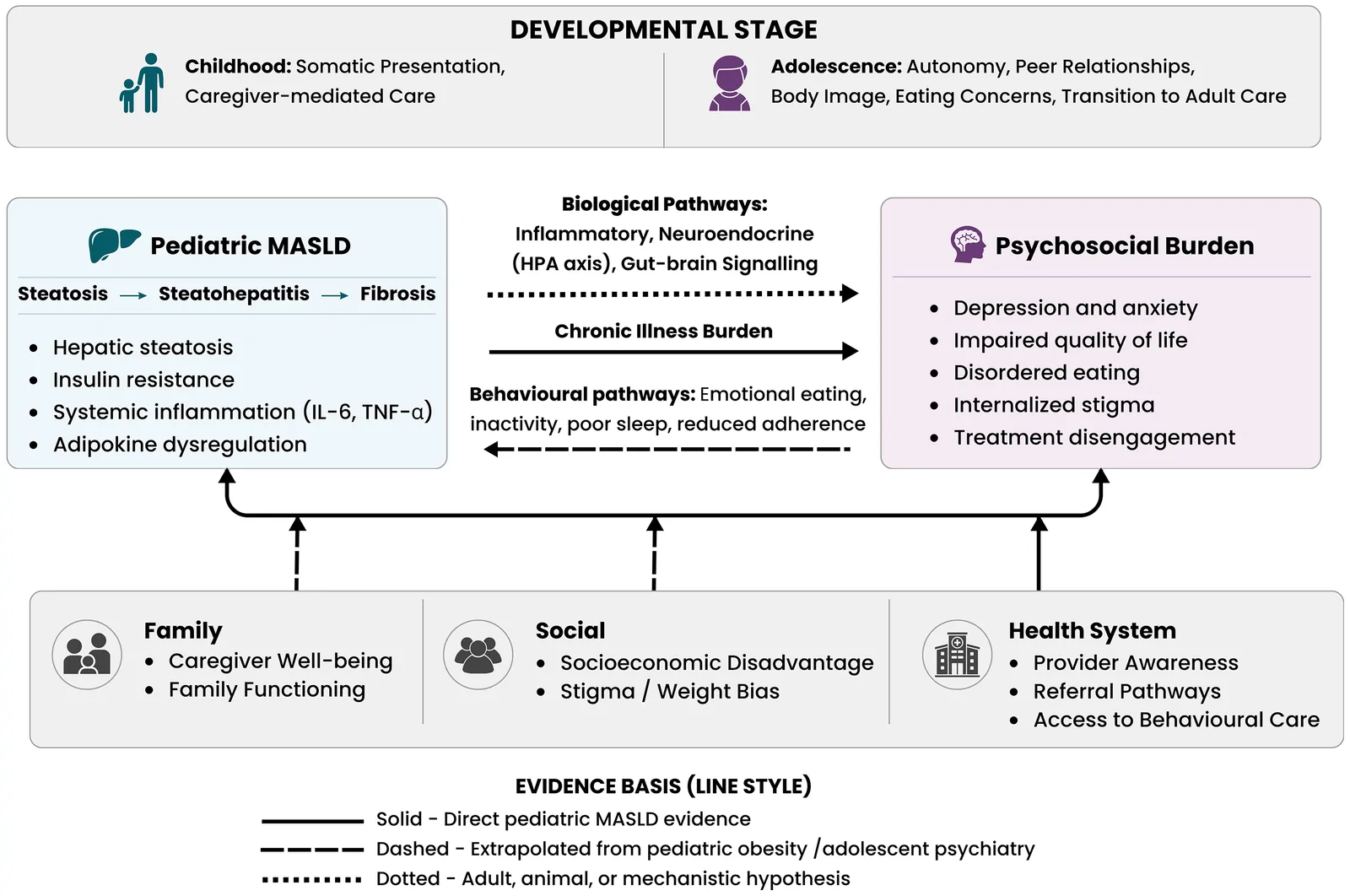
Biopsychosocial model linking pediatric MASLD and psychosocial burden. Three central pathways connect the two: biological (inflammatory and neuroendocrine mechanisms, including the gut–brain axis), chronic illness burden, and *behavioral* (emotional eating, inactivity, poor sleep, reduced adherence), each coded by evidence strength (solid: direct pediatric MASLD evidence; dashed: extrapolated from pediatric obesity or adolescent psychiatry; dotted: adult, animal, or mechanistic evidence). Family, social, and health-system factors act on the system as a whole, and developmental stage (childhood vs. adolescence) shapes how psychosocial burden presents across disease progression (steatosis to steatohepatitis to fibrosis).

Recent estimates suggest that roughly 7–10% of adolescents worldwide meet criteria for MASLD (2), with substantially higher prevalence among children with obesity and other metabolic risk factors. Estimates in pediatric populations range from approximately 5–10% on imaging in general imaging-based cohorts to 30–40% among children with obesity (3, 4). Beyond its long-term hepatic consequences, including steatohepatitis (MASH) and fibrosis, pediatric MASLD may also be associated with impaired health-related quality of life (5). Although clinical encounters often prioritize hepatic and metabolic complications, psychological, *behavioral*, and social factors may shape both the lived experience of MASLD and engagement with its management. This review examines these dimensions within a biopsychosocial framework, including mental health burden, family context, healthcare barriers, and implications for integrated care (**Figure 1**).

Earlier reviews of pediatric MASLD have focused largely on its metabolic and hepatic aspects. Where psychosocial issues have been discussed, the evidence has often been drawn from the broader pediatric obesity literature or extrapolated from adult studies (6). An important uncertainty therefore remains: while children with MASLD appear to experience a considerable psychosocial burden, it is not yet clear whether this represents a distinct consequence of liver disease, or is largely mediated by obesity, coexisting metabolic dysfunction, family context, and social adversity. In this review, we examine the literature through this lens, distinguishing evidence derived directly from pediatric MASLD cohorts from findings extrapolated from pediatric obesity, adult studies, animal models, and proposed mechanistic pathways, highlighting areas of convergence, uncertainty, and clinical relevance.

## Psychological burden of pediatric MASLD

Children and adolescents with MASLD appear to experience a greater burden of emotional and *behavioral* difficulties; however, direct pediatric evidence remains limited and is derived largely from observational studies. Depressive and anxiety disorders have been reported in this population. In a prospective cohort study of 160 adolescents with biopsy-confirmed MASLD followed for approximately 4 years, 8.1% had a diagnosis of clinical depression at baseline and an additional 9.5% developed new-onset depression during follow-up (7). Similarly, 6.3% had anxiety at baseline and 6.7% developed new anxiety (7). The investigators noted that these rates were substantially “higher than expected” compared with general adolescent populations (7). In a small case-control study, children with NAFLD scored significantly higher than age-matched healthy controls on standardized measures of anxiety and depression, including the Multidimensional Anxiety Scale for Children and Children’s Depression Inventory, and had greater internalizing and somatic symptoms on the Child Behavior Checklist (all P < 0.01) (8). However, neither study could determine whether these findings were independent of obesity, family context, socioeconomic adversity, or coexisting metabolic disease.

This psychiatric burden is also reflected in impaired quality of life. Youth with MASLD appear to report poorer health-related quality of life (HRQOL) than peers. In a cohort of 239 children with biopsy-proven NAFLD, total, physical, and psychosocial PedsQL scores were significantly lower than in healthy children, with fatigue, sleep disturbance, and sadness contributing substantially to the impairment (5). Impairment is often most pronounced in emotional and psychosocial domains: children report internalizing symptoms and somatic complaints including fatigue and pain (8). In the study by Kistler et al., quality-of-life scores did not differ according to histological severity. This cross-sectional finding suggests that symptom burden and psychosocial factors may be more closely related to perceived well-being than histological severity, although it does not exclude an association with longer-term liver outcomes (5). Notably, mothers of children with NAFLD also reported lower overall quality of life and poorer relationship-domain scores than mothers of healthy controls, highlighting the wider familial impact of the condition (8). These findings suggest that psychological distress, well-being of the caregivers, and engagement with lifestyle management may interact in a reciprocal manner, although the temporal sequence and direction of these associations have not been established in longitudinal studies. Both studies compared affected children with healthy controls rather than with obesity-matched, liver-disease-free peers, so it remains unclear whether this quality-of-life deficit is specific to MASLD or reflects the accompanying obesity.

Evidence on wider neuropsychiatric and neurodevelopmental comorbidity in pediatric MASLD is still limited. Specific learning disorders have been described more often in children with obesity (9), but these observations cannot be directly extrapolated to MASLD. A single-centre retrospective study reported a higher prevalence of at least one neuropsychiatric diagnosis among children with NAFLD than in a population-based comparison group, with intellectual disability, depression, and autism-spectrum disorder being more commonly reported (10). However, prospective studies are needed before these findings can be interpreted as a liver-specific association. Data on executive function, emotional regulation, and externalizing symptoms in pediatric MASLD remain particularly sparse.

Although direct MASLD-specific evidence remains limited, a developmental and family-*centered* approach is useful in practice. This framing draws on developmental psychopathology, which holds that the same underlying vulnerability can produce different outcomes depending on developmental timing and context, and that different risk pathways can converge on similar psychological presentations (11). Psychological distress may present differently with age: younger children may have somatic complaints, irritability, or *behavioral* change, whereas autonomy, peer relationships, body image, and eating *behavior*s become more relevant during adolescence. Assessment should therefore extend beyond formal diagnoses to include mood and *behavioral* symptoms, eating-related concerns, adherence, parental well-being, and family functioning, as clinically important distress may not always meet diagnostic thresholds (12, 13). This framework is drawn largely from developmental psychiatry and pediatric obesity literature rather than pediatric MASLD cohorts (14, 15). Until MASLD-specific data are available, this framework offers a practical way to identify children and families who may benefit from further support. Protective factors described in the broader pediatric chronic-illness and obesity literature—family cohesion, adaptive coping, and stable caregiving—may buffer this risk in MASLD as well, though this has not been tested directly in affected children and represents an important target for future study.

## Biopsychosocial pathways in pediatric MASLD

The biological links between MASLD and psychosocial health are widely described, but it should be emphasized that almost all of this mechanistic evidence derives from adult cohorts and animal models. Direct pediatric data are scarce, and these pathways are best regarded as biologically plausible rather than established in children. MASLD is characterized by insulin resistance and a pro-inflammatory milieu (for example, elevated IL-6 and TNF-α) that overlaps with pathways implicated in depression (16–19). The associated cytokines, leptin resistance, adipokine dysregulation, and oxidative stress may in turn influence neurotransmission, appetite, and reward pathways (20–26). Whether these processes contribute causally to psychopathology in children with MASLD, however, remains unproven.

The gut–brain axis has been proposed as another potential link. Diets high in fructose, fat, and processed foods may alter gut microbiota composition (27–30), while dysbiosis, intestinal permeability, and systemic inflammation have been associated with anxiety and depressive *behavior*s in animal models and adults (31–33). Alterations in gut hormones such as GLP-1 and ghrelin may also influence mood and appetite, although direct pediatric evidence is lacking. Conversely, psychological stress activates the hypothalamic–pituitary–adrenal axis and may contribute to cortisol excess, insulin resistance, hepatic steatosis, and visceral adiposity (34, 35).

These observations are usually integrated into a proposed feedback loop in which metabolic dysfunction exerts neurobiological effects that predispose to depression and anxiety, while psychological distress promotes *behavior*s, such as poor diet, physical inactivity, and disrupted sleep, that in turn aggravate MASLD. Disrupted sleep may also compound this pathway directly: poor sleep quality and sleep fragmentation have been linked to systemic inflammatory activation in pediatric and adult populations, offering a plausible additional mechanistic route from behavioral disruption to hepatic inflammation (36, 37). Although this model is largely inferred rather than directly demonstrated in children, it carries a clear clinical implication: interventions directed at a single element, such as diet, are unlikely to be adequate unless psychological and social factors are addressed in parallel. It should be noted that many of these mechanisms are shared with adult MASLD; whether childhood onset confers additional or distinct risk remains to be established.

## Social determinants and system-level barriers

MASLD in children is not distributed equally. Socioeconomic status (SES), race and ethnicity, and family environment shape both risk and outcomes. Children from low-SES households face greater exposure to obesogenic environments and often have limited access to care, and the same resource constraints extend to psychosocial care, since families with fewer means may struggle to access mental health services or sustain time-intensive lifestyle programs. Ethnic differences in prevalence are well documented and appear to be driven substantially by genetic variation, notably PNPLA3, in addition to the differences in the social environment rather than by social factors alone (2, 38–41). It should be noted, however, that these prevalence data are largely metabolic in focus and that whether the psychosocial burden differs across these groups has seldom been examined directly.

Psychosocial stigma is a particularly important mediator, and one that is increasingly understood as a driver of poor mental health rather than merely its consequence. Internalized weight bias, wherein affected children direct negative societal attitudes toward themselves, has been shown in young people with obesity to predict depressive symptoms, disordered eating, and avoidance of physical activity, independent of the degree of adiposity (42). This evidence derives from pediatric obesity rather than from MASLD specifically, but it is directly relevant, since framing MASLD as a consequence of personal failure is likely to reinforce the same process; the manner in which the diagnosis is communicated therefore forms part of the clinical burden. Bullying, social isolation, and, in some settings, cultural stigma surrounding mental illness compound this effect and can further delay help-seeking.

Barriers at the level of the health system are equally consequential and, unlike much of the preceding evidence, have been examined directly in pediatric MASLD. A survey of pediatric primary-care providers reported that 92% had little or no familiarity with the condition and less than 3% had knowledge of the relevant guidelines, with knowledge gaps identified as a leading barrier to management (40, 41). Notably, referral to *behavioral* or mental-health services was markedly less common than referral to dietitians in a survey of clinicians managing pediatric obesity (44). Although these findings come from small provider surveys and should be interpreted cautiously, they are consistent with a pattern of delayed recognition and compartmentalized, metabolically focused care in which mental health and social stressors go unaddressed.

## Interventions: screening and multidisciplinary management

Given this burden of psychosocial morbidity in pediatric MASLD, brief and age-appropriate assessment of mood, disordered eating, sleep (including obstructive sleep apnea: bidirectionally linked with both mood and steatosis), and family stressors can be integrated into routine MASLD care, using validated instruments such as the PHQ-A (Patient Health Questionnaire Modified for Adolescents) for depression, a brief anxiety measure such as the SCARED or GAD-7, the PSQ (Pediatric Sleep Questionnaire) or BEARS for sleep, and the PedsQL (Pediatric Quality of Life Inventory) for quality of life. Whether such assessment should be universal, however, remains unsettled, and its value depends on the availability of referral pathways for children who screen positive. Because parental depression and stress impede a child’s disease management, caregivers should be screened and engaged alongside the child (8).

Family-*centered* lifestyle intervention remains the cornerstone of management (45, 46), combining nutrition, structured activity, and *behavioral* techniques, with cognitive-*behavioral* components that improve both weight and emotional outcomes (47, 48). It should be emphasized, however, that this evidence derives largely from pediatric obesity rather than from MASLD-specific trials, and the hepatic benefit in children is for the most part inferred. An important and under-recognized tension attends these weight-*centered* approaches: loss-of-control and binge eating are common in children with obesity, and a weight- and food-focused frame may, in vulnerable children, entrench disordered eating rather than relieve it (49). Screening for disordered eating should therefore precede and accompany any structured weight intervention, and an established eating disorder should be co-managed rather than subordinated to a weight target.

Where a formal psychiatric disorder is present, it should be treated on its own merits, with cognitive *behavioral* therapy (CBT), family therapy, or pharmacotherapy as indicated. Disorder-specific data in MASLD are lacking; evidence from pediatric gastroenterology suggests that integrating *behavioral* health into subspecialty care may improve psychosocial outcomes, although implementation evidence remains limited (50, 51). Where multidisciplinary teams spanning hepatology, nutrition, psychology, and social work are not feasible, co-located services or telehealth can approximate them, and embedding a mental health screen into routine obesity visits, along with dissemination of guidelines, may help close the recognition gap identified above (43).

Pharmacological options are now available for adolescent obesity and raise their own psychosocial considerations. GLP-1 receptor agonists are increasingly used in adolescent obesity, with semaglutide approved from age 12, and reduce body weight, hepatic transaminases, and weight-related impairment of quality of life (52). An early signal of possible suicidal ideation was not borne out on subsequent review (53), and the corresponding labeling warning has since been removed by the Food and Drug Administration (FDA) (54). However, it is important to note that MASLD-specific data on mood and eating-related effects in children remain scarce, so these domains warrant active monitoring during treatment.

Taken together, the evidence, though drawn largely from pediatric obesity and adult NAFLD rather than from pediatric MASLD itself, favors an integrated model in which metabolic and psychological care are delivered together. This integration should extend to the move from pediatric to adult services, a transition at which many young people are lost to follow-up. Transition planning should begin in early adolescence and progressively build self-management skills, including understanding of liver and metabolic status, treatment goals, lifestyle adherence, and psychological support when relevant. This warrants a planned, structured handover with continued mental health screening, since transition-specific evidence in MASLD is lacking and the case rests on other pediatric chronic diseases (55). Addressing the psychosocial burden of pediatric MASLD means treating not only the liver but the child and family as a whole.

## Discussion

Pediatric MASLD is best understood not as a metabolic disorder alone but as a biopsychosocial condition, in which hepatic, psychological, and social processes reinforce one another. Anxiety, depression, disordered eating, and impaired quality of life are common and in turn shape adherence, lifestyle, and disease course. The central unresolved question, however, is how much of this burden is specific to the liver disease. Whether the psychiatric morbidity is intrinsic to MASLD or largely a correlate of the accompanying obesity, chronic illness burden, socioeconomic disadvantage, family dysfunction, poor sleep quality, and insulin resistance cannot be determined from the predominantly cross-sectional evidence, and few studies have examined whether liver disease severity itself predicts psychological symptoms. The direction of causation is equally uncertain, since the inflammatory and gut–brain pathways commonly invoked, though biologically plausible, rest largely on adult and experimental work rather than on direct demonstration in the pediatric age group.

Several more specific controversies follow. The most clinically immediate is the tension between the weight-*centered* interventions that anchor MASLD management and the risk that a weight- and food-focused frame may entrench disordered eating in vulnerable children (49); how best to navigate this is not established. It also remains unclear whether routine psychosocial screening is feasible and cost-effective in real-world pediatric and primary-care settings, given documented gaps in provider awareness and access to services (43, 44). The neuropsychiatric safety of GLP-1 receptor agonists is better resolved than previously thought: early post-marketing concerns regarding suicidal ideation were not supported by subsequent regulatory review (53, 54). The more pertinent gap is the scarcity of pediatric MASLD-specific data on mood and eating-related outcomes during treatment.

These uncertainties define the research agenda: longitudinal pediatric studies that link psychosocial intervention to both metabolic and mental-health outcomes; screening instruments validated in this population; pragmatic trials of integrated care models; and implementation research addressing how such models, and the transition to adult care, can be sustained in real-world practice (8). Biomarkers that identify children at greatest psychiatric risk, and digital or precision-medicine approaches tailored to developmental stage and risk profile, represent further directions that remain largely unexplored in pediatric MASLD.

## Clinical implications

For practice, the implication is clear even if the evidence is incomplete. Brief psychosocial assessment using validated instruments should be considered within routine metabolic care, particularly where clear assessment, safety, and referral pathways are available (**Table 1**); screening for disordered eating may be incorporated alongside weight-focused management, ideally delivered within a family-*centered* framework; and, where feasible, care should be organized through teams that bring together hepatology, endocrinology, nutrition, and *behavioral* health, with attention to continuity as adolescents transition to adult services. Even in resource-constrained settings, attention to caregiver well-being, sleep-related concerns, and practical barriers to engagement may be valuable. By treating mind and body together, clinicians may improve not only liver health but the long-term resilience of these children.

**Table 1:** Suggested domains and instruments for psychosocial and mental-health assessment in pediatric MASLD.

Domain	Suggested instrument(s)	Typical age range (yr)/validated groups	Frequency	Positive screen: interpretation & action	Validation in pediatric MASLD
Depression	PHQ-A; PHQ-9 modified for adolescents	Adolescents; commonly 11–17	At diagnosis, then annually or when clinically indicated	Score above cutoff → clinical assessment; positive self-harm/ideation item → same-day safety assessment and referral	Not validated in MASLD; validated in adolescent primary care
Anxiety	SCARED; GAD-7	SCARED 8–18; GAD-7 in adolescents	At diagnosis; repeat if concern	Score above cutoff → further assessment/referral	Not validated in MASLD
Disordered eating	SCOFF; ChEAT	SCOFF in adolescents; ChEAT 8–13	Before and during any weight-focused intervention	Positive → eating-disorder assessment and co-management; urgent referral if medically unstable	Not validated in MASLD; interpret cautiously in higher-weight youth
Quality of life	PedsQL	2–18 (self-report ≥5)	Baseline and to monitor	Low score → structured review of physical and psychosocial impairment	Used in pediatric NAFLD studies; not validated as a screen
Weight-bias internalization	WBIS-Y	Adolescents	As indicated (evident stigma or low self-esteem)	High score → address illness framing; offer psychological support	Not validated in MASLD; primarily a research tool
Sleep/OSA	Pediatric Sleep Questionnaire (PSQ); BEARS	2–18	At assessment	Positive → sleep evaluation; polysomnography if indicated	Not validated in MASLD; OSA associated with MASLD
Family/caregiver	Caregiver PHQ-2/PHQ-9; brief family-functioning measure	Caregiver	At intake or when family stressors are evident	Positive → discuss caregiver support needs; referral where feasible	Not applicable (caregiver-directed)

None of these instruments is diagnostic or has been validated specifically in pediatric MASLD; each is validated in general pediatric, primary-care, sleep, mental-health, or obesity populations and is proposed here as a pragmatic tool pending MASLD-specific validation. Suggested frequencies are pragmatic rather than evidence-based. Instruments should be selected according to the child’s age, language, and locally validated cutoffs, and screening is appropriate only where a referral pathway is available. Any positive screen for suicidal ideation requires immediate safety assessment. BEARS, Bedtime–Excessive daytime sleepiness–Awakenings–Regularity–Snoring screen; ChEAT, Children’s Eating Attitudes Test; GAD-7, Generalized Anxiety Disorder-7; OSA, obstructive sleep apnea; PedsQL, Pediatric Quality of Life Inventory; PHQ, Patient Health Questionnaire; PSQ, Pediatric Sleep Questionnaire; SCARED, Screen for Child Anxiety Related Disorders; SCOFF, Sick-Control-One stone-Fat-Food questionnaire; WBIS-Y, Weight Bias Internalization Scale for Youth.

## Conclusion

Three messages may be worth considering for practice, while acknowledging that direct pediatric MASLD-specific evidence for each remains limited. Brief, validated screening for depression, anxiety, and disordered eating may be a useful component of routine MASLD follow-up rather than a purely optional adjunct (**Table 1**). It may be prudent for weight-focused counselling to be preceded by screening for disordered eating, given the risk that a weight-*centered* frame could itself precipitate it. Caregiver well-being might also be considered part of the child’s care, since parental distress has been associated with impeded disease management. Whether these measures improve outcomes specific to MASLD, rather than obesity more broadly, remains for future research to confirm.
